# Quantitative analyses of the global proteome and phosphoproteome reveal the different impacts of propofol and dexmedetomidine on HT22 cells

**DOI:** 10.1038/srep46455

**Published:** 2017-04-18

**Authors:** Honggang Zhang, Juan Ye, Zhaomei Shi, Chen Bu, Fangping Bao

**Affiliations:** 1 Department of Anesthesiology, the First Affiliated Hospital, College of Medicine, Zhejiang University, Hangzhou, 310003, China; 2 Department of Pediatrics, the First Affiliated Hospital, College of Medicine, Zhejiang University, Hangzhou, 310003, China; 3 Jingjie PTM Biolab (Hangzhou) Co. Ltd., Hangzhou 310018, China

## Abstract

Propofol and dexmedetomidine are both commonly used anaesthetics. Although they employ two different mechanisms to induce anaesthesia, both compounds influence the hippocampus and the HT22 cell line. HT22 cells are broadly used in neurobiological research. In this study, we assessed the effects of propofol and dexmedetomidine on signalling in HT22 cells. Using the SILAC (stable isotope labelling with amino acids in cell culture) labelling technique, IMAC (immobilized metal affinity chromatography) enrichment and high-resolution LC-MS/MS (liquid chromatography tandem mass spectrometry) analysis, we investigated the quantitative proteome and phosphoproteome in HT22 cells treated with propofol or dexmedetomidine. In total, 4,527 proteins and 6,824 phosphosites were quantified in cells treated with these two anaesthetics. With the assistance of intensive bioinformatics, the propofol and dexmedetomidine treatments were shown to induce distinct proteome and phosphoproteome profiles in HT22 cells. Consistent with our bioinformatics analysis, dexmedetomidine had a smaller effect than propofol on cell survival. These findings deepen our understanding of drug-induced anaesthesia.

Not surprisingly, the development of anaesthesia has substantially contributed to modern surgery. However, the potential side effects of anaesthetics and the increasing demand for better medical services have prompted researchers to obtain a deeper understanding of the mechanisms of commonly used anaesthetics^1^. Due to its antioxidant, anti-inflammatory and bronchiectasis properties^2^, propofol (2,6-diisopropylphenol) has been widely used to induce and maintain general anaesthesia since its discovery in 1977^3^. At the molecular level, propofol directly activates the gamma-amino butyric acid A (GABAA) receptor^4^,^5^ but slightly suppresses the activity of the N-methyl-D-aspartate (NMDA) receptor^6^. Although propofol is remarkably safe in clinical use, it still introduces has complications, including irregular heart rates, low blood pressure, pain upon injection and stopping of breath^2^. Dexmedetomidine is a specific agonist of α_2_-receptors and has been used as an adjunct to anaesthesia, based on its sedative, analgesic and antishivering properties^7^. The differences between the mechanisms of propofol and dexmedetomidine have allowed these compounds to be used in combination in clinical practice to achieve better anaesthetic effects^8^,^9^.

The hippocampus is an important component of the brain that plays a key role in consolidating information from the short-term memory to the long-term memory. The hippocampus is known to participate in general anaesthesia^10^, and therefore, general anaesthesia may inhibit learning and memory^11^. HT22 cells are a sub-line derived from parental HT4 cells, which were originally immortalized from a primary mouse hippocampal neuron culture^12^, and they have been widely used as a cell model in neurobiological studies of Alzheimer’s disease, Parkinson’s disease and the effects of general anaesthesia^13^,^14^,^15^,^16^,^17^. Both propofol and dexmedetomidine influence the hippocampus and the HT22 cell line. For example, propofol modulates the activation and desensitization of GABAA receptors in cultured murine hippocampal neurons^18^ and inhibits angiotensin II-induced HT22 cell apoptosis^19^. Dexmedetomidine confers neuroprotective effects against ischaemia and hypoxia upon the hippocampus^20^,^21^. Additionally, both propofol and dexmedetomidine impair the induction of hippocampal long-term potentiation^22^,^23^. Therefore, HT22 cells are a good model to study the effects of propofol and dexmedetomidine on cell signalling. Although some processes and signalling pathways have been reported to be related to dexmedetomidine and/or propofol treatments, the comprehensive mechanisms of these two anaesthetics are still unclear, particularly at the proteomic level.

The liquid chromatography-tandem mass spectrometry (LC-MS/MS)-based proteomic analysis method has emerged as a powerful tool to study signal transduction due to its high throughput capacity and high accuracy^24^,^25^,^26^. Although some studies have employed 2D-electrophoresis to investigate the propofol-induced alterations in the proteome and phosphoproteome in the rat hippocampus, the inherent disadvantages of the 2D system restrict its potential utility in discovery and analysis of the global proteome^27^,^28^,^29^. In this work, we comprehensively compared the proteomes and phosphoproteomes of control, propofol- and dexmedetomidine-treated HT22 cells using LC-MS/MS-based methods and identified 5,151 proteins and 7,040 phosphosites. According to the integrative bioinformatics analysis, diverse processes, including vesicle transport, chromosome structure, oxidative phosphorylation and signal transduction, were regulated by the dexmedetomidine and propofol treatments to different extents. These findings revealed the impacts of dexmedetomidine and propofol on intracellular signal transduction, which might facilitate the clinical applications of dexmedetomidine and propofol.

## Results

### Strategy used for the quantitative proteome and phosphoproteome analyses

**Figure 1:**
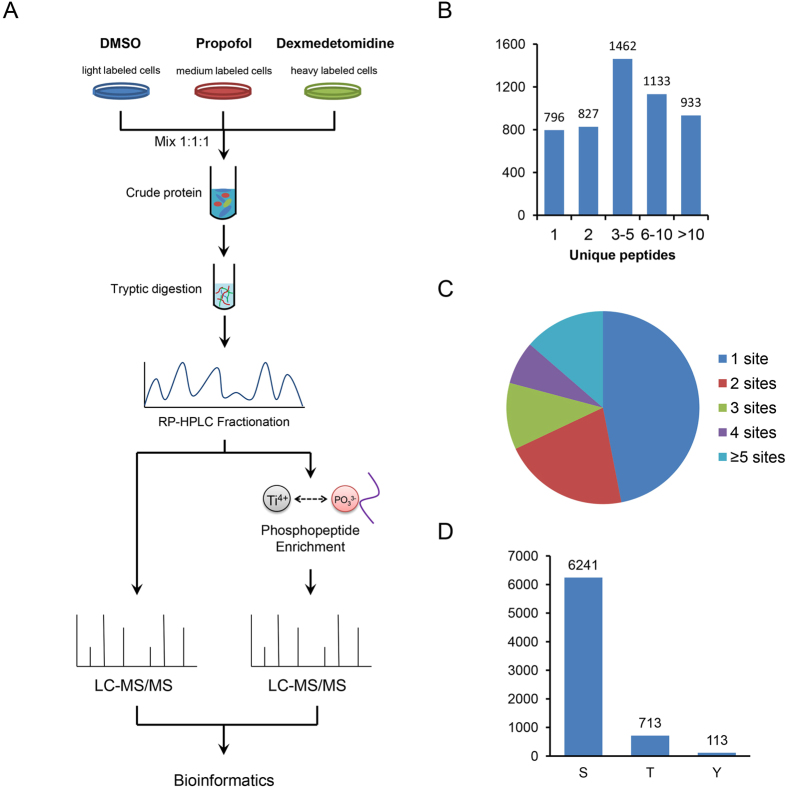
Overview of the proteomes and phosphoproteomes of propofol- and dexmedetomidine-treated HT22 cells. (**A**) Systematic workflow of the quantitative proteome and phosphoproteome analyses. (**B**) Distribution of the identified proteins based on the number of unique peptides. (**C**) Pie chart showing the distribution of the number of phosphosites identified per protein. (**D**) Bar chart showing the numbers of serine, threonine and tyrosine phosphosites.

Although some mechanisms of propofol and dexmedetomidine have been reported, their effects on intracellular signal transduction remain to be elucidated, particularly at the proteome level. In this study, we combined the stable isotope labelling with amino acids in cell culture (SILAC) labelling method, HPLC fraction, immobilized metal affinity chromatography (IMAC) enrichment, high resolution Orbitrap mass spectrometry and bioinformatics analyses for the systematic quantification of the proteomes and phosphoproteomes in control, propofol-treated and dexmedetomidine-treated HT22 cells. The integrated workflow is shown in Fig. 1A. Using this method, we identified 5,151 proteins in HT22 cells, of which at least 2 unique peptides were identified for 4,355 proteins (Fig. 1B). A total of 7,040 phosphosites in 2,670 proteins were identified in our phosphoproteome analysis, and nearly half of these phosphoproteins had only one identified phosphosite (Fig. 1C). The 7,040 phosphosites included 6,241 (89%) serine, 713 (10%) threonine, and 113 (2%) tyrosine phosphorylation sites (Fig. 1D).

### Impacts of dexmedetomidine and propofol on the global proteome of HT22 cells

In this study, 4,527 of the 5,151 identified proteins were quantified. With a fold-change threshold > 1.5, 321 and 276 proteins were up- and down-regulated, respectively, in propofol-treated HT22 cells, whereas 147 and 158 proteins were up- and down-regulated, respectively, in dexmedetomidine-treated HT22 cells. All these data are presented in Supplementary Table S1. SDS-PAGE and Western blots were performed to validate the results of our quantitative SILAC analysis (Figure S1).

**Figure 2:**
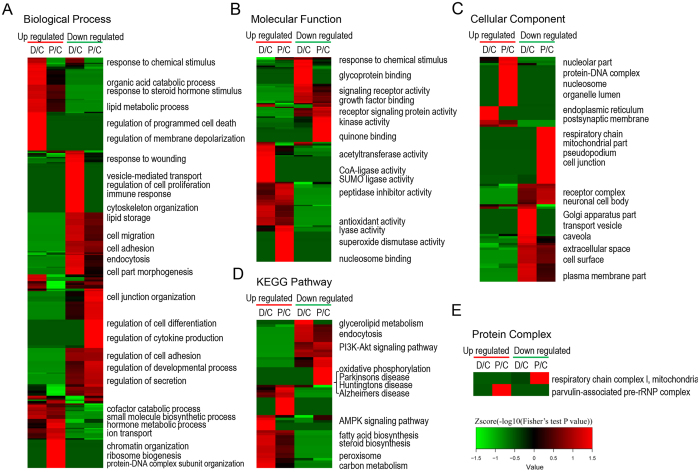
Functional enrichment-based clustering analysis of the quantified proteomics datasets from propofol- and dexmedetomidine-treated HT22 cells. (**A**) Biological process analysis. (**B**) Molecular function analysis. (**C**) Cellular component analysis. (**D**) KEGG pathway analysis. (**E**) Protein complex analysis.

A Gene Ontology (GO) enrichment-based clustering analysis was performed to characterize the functions of the differentially expressed proteins in the propofol- and dexmedetomidine-treated HT22 cells (Fig. 2). In the biological process category (Fig. 2A), response to chemical stimulus, the regulation of programmed cell death and the regulation of membrane depolarization were up-regulated in the dexmedetomidine-treated but not propofol-treated cells, whereas the chromatin organization, ribosome biogenesis and protein-DNA complex subunit organization processes were up-regulated in the propofol-treated but not dexmedetomidine-treated cells. In addition, cofactor catabolic process and small molecule biosynthetic process were up-regulated in both dexmedetomidine- and propofol-treated cells. In contrast, extracellular matrix organization, regulation of cell proliferation and immune response were down-regulated in dexmedetomidine-treated cells; the regulation of cell differentiation, cellular respiration and the regulation of cytokine production were down-regulated in propofol-treated cells; and lipid storage and cell adhesion processes were down-regulated in cells treated with each anaesthetic. In the molecular function category (Fig. 2B), acetyltransferase activity, sterol binding, CoA-ligase activity and SUMO ligase activity were up-regulated in response to the dexmedetomidine treatment, whereas lyase activity, superoxide dismutase activity and nucleosome binding were up-regulated in response to the propofol treatment. Moreover, peptidase inhibitor activity and anion transmembrane transporter activity were up-regulated in propofol- and dexmedetomidine-treated cells. The results of the cellular component-based clustering analysis are shown in Fig. 2C. Dexmedetomidine-treated cells were enriched in proteins located in the endoplasmic reticulum and postsynaptic membrane, whereas propofol-treated cells were enriched in proteins located in the nucleoli, nucleosome and organelle lumen. In contrast, the down-regulated proteins in the dexmedetomidine and propofol-treated cells were primarily located in the mitochondria and transport vesicles, respectively. In addition, both dexmedetomidine and propofol induced the down-regulation of some membrane and extracellular proteins. Although some processes were regulated by both dexmedetomidine and propofol, the two drugs still had some different effects on HT22 cells.

Enrichment-based clustering analyses were performed using the Kyoto Encyclopedia of Genes and Genomes (KEGG) database to profile the cellular pathways regulated by the dexmedetomidine or propofol treatments (Fig. 2D). The AMPK and PPAR signalling pathways were up-regulated in the dexmedetomidine-treated cells; ribosome and mucin type O-glycan biosynthesis were up-regulated in the propofol-treated cells; and steroid biosynthesis, fatty acid biosynthesis and the peroxisome were up-regulated in response to treatment with the two anaesthetics. On the other hand, glycerolipid metabolism was down-regulated in response to dexmedetomidine, oxidative phosphorylation and neurodegenerative disease signalling were down-regulated in response to propofol, and PI3K-Akt signalling was inhibited in cells treated with dexmedetomidine or with propofol. According to the subsequent analysis of the protein complexes, the parvulin-associated pre-rRNP complex was up-regulated, but respiratory chain complex 1 was down-regulated in propofol-treated cells (Fig. 2E).

**Figure 3:**
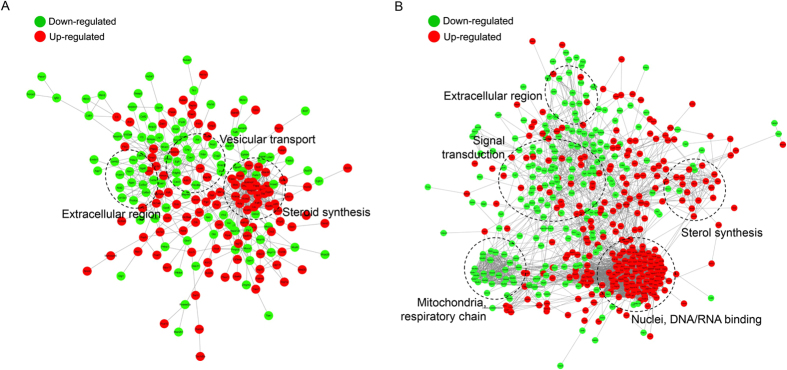
Analyses of the protein-protein interaction networks for the differentially expressed proteins in dexmedetomidine-treated (**A**) and propofol-treated (**B**) HT22 cells.

We then established the protein-protein interaction networks for the differentially expressed proteins observed following the dexmedetomidine or propofol treatment (Fig. 3). Consistent with the clustering analyses, proteins related to the extracellular region and steroid synthesis were altered in response to treatment with both anaesthetics, whereas vesicular transport, the mitochondrial respiratory chain and DNA/RNA binding were differentially regulated by the dexmedetomidine or propofol treatment.

### Impacts of dexmedetomidine and propofol on the phosphoproteome of HT22 cells

Phosphorylation plays important roles in signal transduction and the process of anaesthesia^30^,^31^. After IMAC enrichment and the LC-MS/MS analysis, 7,040 phosphosites in 2,670 proteins were identified, and 6,824 phosphosites in 2,599 proteins were quantified (Supplementary Table S2). We then normalized the phosphoproteome data to the results of the global proteome analysis (Supplementary Table S3). The quantitative ratios of the proteome and phosphoproteome upon dexmedetomidine or propofol treatment were compared and are shown in Figure S2. Based on a fold-change threshold >1.5, the dexmedetomidine treatment up-regulated 453 phosphosites in 352 proteins and down-regulated 671 phosphosites in 441 proteins, whereas the propofol treatment up-regulated 498 phosphosites.

**Figure 4:**
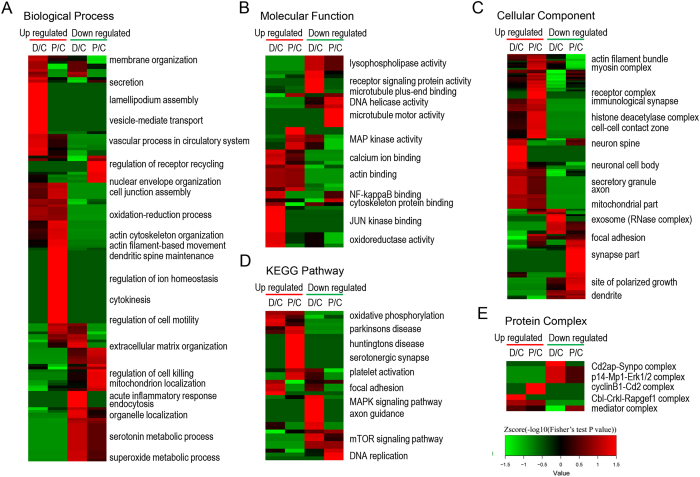
GO enrichment-based clustering analysis of the quantified phosphoproteome. (**A**) Biological process analysis. (**B**) Molecular function analysis. (**C**) Cellular component analysis. (**D**) KEGG pathway analysis. (**E**) Protein complex analysis.

A GO enrichment-based clustering analysis was performed to characterize the functions of phosphosites that were differentially regulated by the dexmedetomidine or propofol treatment. As shown in Fig. 4A, the biological process analysis revealed that the membrane organization, vesicle-mediated transport, secretion-related processes; extracellular matrix organization; acute inflammatory response; and endocytosis process were up- and down-regulated in dexmedetomidine-treated cells, respectively. Processes related to dendritic spine maintenance, the regulation of ion homeostasis, cytokinesis, the regulation of cell motility and protein-DNA complex subunit regulation, nucleus organization, mitochondrion localization were up- and down-regulated in propofol-treated cells, respectively. In addition, oxidation-reduction process and superoxide metabolic process were up- and down-regulated by both drugs, respectively. For the molecular function cluster (Fig. 4B), JUN kinase binding and NF-kappaB binding were up-regulated by the dexmedetomidine treatment, whereas MAP kinase activity was up-regulated by the propofol treatment, suggesting that these two anaesthetics affect different kinase-mediated signalling pathways. In the cellular component category (Fig. 4C), the proteins that were differentially expressed proteins in response to the dexmedetomidine and propofol treatments were mainly located in the ribosome, endomembrane system, exosome, neuronal cell body and cytoskeleton, chromosome, and synapse. In addition, both anaesthetics regulated proteins located in the mitochondria and dendrites.

Then, KEGG clustering was performed to characterize the alterations in signalling pathways induced by the dexmedetomidine and propofol treatments (Fig. 4D). The changes observed in some signalling pathways, including oxidative phosphorylation, ribosomes and the MAPK signalling pathway, were consistent with the results of the GO enrichment analysis. Interestingly, Parkinson’s disease and Huntington’s disease pathways were up-regulated in propofol-treated cells, whereas the axon guidance pathway was down-regulated in the dexmedetomidine-treated cells, suggesting that these two drugs may affect neuronal functions through different pathways. In addition, our analysis of the protein complexes revealed that respiratory chain complex 1 was up-regulated in both dexmedetomidine- and propofol-treated cells, consistent with previous analyses (Fig. 4E).

**Figure 5:**
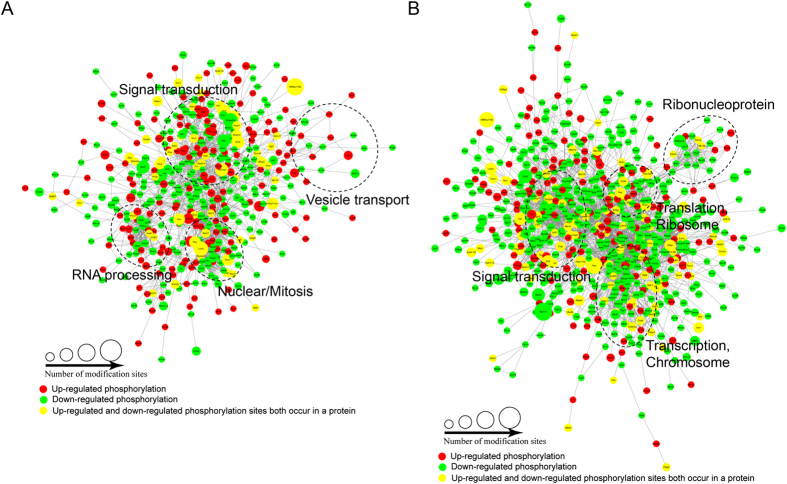
Analyses of the protein-protein interaction networks for differentially expressed phosphorylated proteins in dexmedetomidine-treated (**A**) and propofol-treated (**B**) HT22 cells.

Protein-protein interaction networks were established based on the proteins whose phosphosites were regulated by the dexmedetomidine or propofol treatment to further characterize the impacts of dexmedetomidine and propofol on the proteome of HT22 cells (Fig. 5). Consistent with the previous clustering analysis, the two anaesthetics affected distinct processes, such as vesicle transport, chromosome structure and signal transduction, at the phosphorylation level.

### Dexmedetomidine has a smaller effect than propofol on cell survival

**Figure 6:**
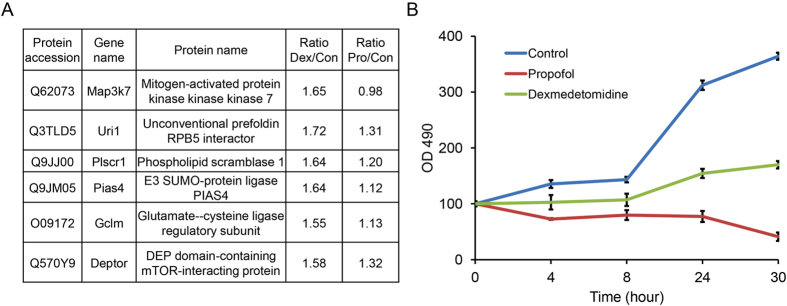
Different effects of dexmedetomidine and propofol on cell growth. (**A**) Changes in the abundance of some apoptosis-related proteins observed in dexmedetomidine- and propofol-treated HT22 cells. (**B**) The MTT assay was used to determine the growth curves of control, propofol- and dexmedetomidine-treated HT22 cells.

Based on our bioinformatics analysis, dexmedetomidine and propofol had different effects on the regulation of programmed cell death (Fig. 2A). For example, the expression levels of some apoptosis-related proteins, such as Uri1, Pias4 and Gclm, were significantly up-regulated by dexmedetomidine, but not propofol, as shown in our proteomic results (Fig. 6A). Because Uri-1, Pias4 and Gclm have been reported to negatively regulate apoptosis^32^,^33^,^34^, we concluded that the up-regulation of these proteins in the dexmedetomidine-treated HT22 cells suggests that dexmedetomidine, but not propofol, reduces neuronal apoptosis. We assessed the growth curves of cells treated with each anaesthetic to confirm this hypothesis. Based on the results of MTT assays, dexmedetomidine had a smaller effect than propofol on HT22 cell growth (Fig. 6B), consistent with the results of our bioinformatics analysis and previous reports^35^,^36^.

## Discussion

Although dexmedetomidine and propofol are widely used anaesthetics in surgery, their effects on hippocampal cells at the proteome level remain elusive. In this study, we systematically analysed the proteomes and phosphoproteomes of dexmedetomidine- and propofol-treated HT22 cells. The two anaesthetics have common and distinct influences on various cellular functions and processes, such as oxidative phosphorylation, chromosome structure regulation, vesicle transport processes and signal transduction.

### Effects of dexmedetomidine and propofol on oxidative phosphorylation

The mitochondria are well known to participate in neurodegenerative diseases through their roles in regulating apoptosis and oxidative stress^37^,^38^. At the proteome level, propofol, but not dexmedetomidine, reduced the expression levels of proteins involved in the mitochondrial respiratory chain (Fig. 2C). Consistent with this finding, our KEGG analysis revealed that pathways associated with neurodegenerative diseases and oxidative phosphorylation signalling were down-regulated in propofol-treated HT22 cells (Fig. 2D). Many NADH dehydrogenases, such as Ndufs4, Ndufs5, Ndufs6, and Ndufa8, were down-regulated by the propofol treatment (Fig. 3B), and the inhibition of mitochondrial NADH dehydrogenase has been reported to play a major role in Parkinson’s disease^39^,^40^. In addition, NADH dehydrogenase has a significant role in synaptic NMDA receptor activity^41^, consistent with the observation that propofol suppressed the activity of the NMDA receptor^6^.

In contrast to the proteome-level data, our phosphoproteome analysis revealed that both dexmedetomidine and propofol may affect oxidative phosphorylation (Fig. 4). The phosphorylation of Y142 in Ndufa8 and Y109 in Ndufc2 was increased in HT22 cells treated with each anaesthetic compared to control cells. Although the functions of these two tyrosine phosphosites are still not clear, the results suggested that dexmedetomidine and propofol may affect NADH dehydrogenase by modulating protein phosphorylation. Based on our data, propofol may have a stronger influence than dexmedetomidine on NADH dehydrogenase and neuron and synapse function.

### Effects of dexmedetomidine and propofol on transcription and chromosome assembly

Transcription and chromosome assembly were also differentially regulated by dexmedetomidine and propofol. At the proteome level, the propofol treatment increased the expression of some proteins related to chromatin organization (Fig. 2A). For example, the levels of some histone proteins, particularly histone H1 proteins, and transcriptional regulators, including Baz1b, Sap130, Top1 and Wac, were increased in propofol-treated cells. Chromatin has a proven role in neural development^42^. The up-regulated expression of histone H1 has been reported to be related to Alzheimer’s disease^43^, a neurodegenerative disease, and histone H1 dynamics have important roles in neural dysfunction^44^.

At the phosphoproteome level, the phosphorylation of diverse transcription factors and chromosomal proteins were down-regulated in propofol-treated cells, including T367 in EZH2. The phosphorylation of T367 in EZH2 plays a role in TNF/p38a/Polycomb signalling^45^, suggesting that propofol may influence epigenetic regulation.

### Effects of dexmedetomidine and propofol on GABAA and α2-receptor signalling

According to previous reports, dexmedetomidine directly activates the GABAA receptor and then induces the reorganization of the actin cytoskeleton in neurons into ring structures through Rho/ROCK signalling^46^. Rho proteins are activated by GEFs (Rho guanine nucleotide exchange factors) and inhibited by GAPs (Rho GTPase-activating proteins)^47^. Based on our bioinformatics analysis, propofol, but not dexmedetomidine, significantly regulated the organization of the actin cytoskeleton (Fig. 4A). Thirty-one phosphosites in Rho regulators were significantly regulated in propofol-treated cells, whereas only 22 phosphosites in Rho regulators were significantly regulated in dexmedetomidine-treated cells (Supplementary Table S3), suggesting that propofol had a stronger effect on the organization of the actin cytoskeleton. On the other hand, dexmedetomidine has been reported to attenuate oxidative stress by activating the α2-receptor^48^, consistent with the results of our analysis showing that dexmedetomidine, but not propofol, significantly regulated oxidoreductase activity (Fig. 4B).

## Materials and Methods

### SILAC labelling and drug treatment of HT22 cells

The control HT22 cells were labelled as light (K0R0), medium (K4R6) and heavy (K8R10) using a SILAC Protein Quantification Kit (Pierce, Thermo Fisher Scientific) according to the manufacturer’s instructions. Briefly, the cell line was cultured in Dulbecco’s Modified Eagle’s Medium supplemented with 10% foetal bovine serum for more than six generations before being harvested to achieve greater than 97% labelling efficiency. Then, the cells were further expanded in SILAC media to the desired number of cells (~5 × 10^8^) in fifteen flasks (Φ = 15 cm). The cells labelled “medium” were then treated with 500 μg/mL propofol, and the cells labelled “heavy” were treated with 10 μg/mL dexmedetomidine hydrochloride for 8 hours. Then, the cells were washed twice with ice-cold PBS and harvested as previously described^49^.

### Trypsin digestion and HPLC fractionation

Trypsin (Promega) was added to the protein solution at ratio of trypsin to protein of 1:50 (w/w), and the solution was digested at 37 °C for 16 hours. Dithiothreitol (DTT) was then added to a final concentration of 5 mM, followed by incubation at 37 °C for 1 hour. Then, iodoacetamide (IAA) was added to a final concentration of 15 mM to alkylate proteins and incubated at room temperature in the dark for 30 min. The alkylation reaction was quenched with 30 mM cysteine (final concentration) and incubated at room temperature for an additional 30 min. Next, trypsin was added to the solution again at a ratio of trypsin to protein of 1:100 (w/w) and incubated at 37 °C for 4 hours to complete the digestion cycle.

The sample was fractionated using high pH reverse-phase HPLC on an Agilent 300Extend C18 column (5 μm particles, 4.6 mm ID and 250 mm long). Briefly, peptides were first separated into 80 fractions with a gradient of 2% to 60% acetonitrile in 10 mM ammonium bicarbonate, pH 10, over 80 min. Then, the peptides were combined into 18 fractions and dried by vacuum centrifugation.

### IMAC enrichment of phosphopeptides

First, peptide mixtures were incubated with an IMAC microsphere suspension with vibration. The IMAC microspheres with enriched phosphopeptides were collected by centrifugation, and the supernatant was removed. The IMAC microspheres were sequentially washed with 50% acetonitrile (ACN)/6% trifluoroacetic acid (TFA) and 30% ACN/0.1% TFA to remove the non-specifically adsorbed peptides. An elution buffer containing 10% NH_4_OH was added to the suspension and vibrated to elute the enriched phosphopeptides from the IMAC microspheres. The supernatant containing the phosphopeptides was collected and lyophilized for the LC-MS/MS analysis.

### LC-MS/MS analysis

Peptides were dissolved in solvent A (0.1% formic acid (FA) in 2% ACN) and directly loaded onto a reverse-phase pre-column (Acclaim PepMap 100, Thermo Fisher Scientific). Peptides were separated using a reverse-phase analytical column (Acclaim PepMap RSLC, Thermo Fisher Scientific) with a linear gradient of 4–22% solvent B (0.1% FA in 98% ACN) for 50 min, 22–35% solvent B for 12 min and 35–85% solvent B for 4 min, then holding at 85% for the last 3 min at a constant flow rate of 300 nL/min on an EASY-nLC 1000 UPLC system. The resulting peptides were analysed on a Q Exactive Plus hybrid quadrupole-Orbitrap mass spectrometer (Thermo Fisher Scientific).

The peptides were subjected to a nanospray ionization (NSI) source followed by tandem mass spectrometry (MS/MS) in Q Exactive Plus (Thermo Fisher Scientific) coupled online to the ultraperformance liquid chromatograph (UPLC). Intact peptides were detected by the Orbitrap at a resolution of 70,000. Peptides were selected for MS/MS using a normalized collision energy (NCE) of 30; ion fragments were detected by the Orbitrap at a resolution of 17,500. A data-dependent procedure that alternated between one MS scan followed by 20 MS/MS scans was applied for the top 20 precursor ions, with a 15.0 s dynamic exclusion. The electrospray voltage applied was 2.0 kV. MS1 spectra were obtained with an automatic gain control (AGC) target of 3 × 10^6^ ions and a maximum injection time of 50 ms, and MS2 spectra were acquired with an AGC target of 5 × 10^4^ ions and a maximum injection time of 200 ms. For MS scans, the m/z scan range was 350 to 1800. The mass spectrometry and proteomics data have been deposited into the ProteomeXchange Consortium via the PRIDE^50^ partner repository with the dataset identifier PXD004687.

### Database search and bioinformatics analysis

The resulting MS/MS data were processed using MaxQuant with the integrated Andromeda search engine (v.1.4.1.2). Tandem mass spectra were searched against the *SwissProt_mouse* database concatenated with a reverse decoy database. Trypsin/P was specified as the cleavage enzyme and up to 2 missed cleavages, 5 modifications per peptide and 5 charges were allowed. Mass error was set to 10 ppm for precursor ions and 0.02 Da for fragment ions. The carbamidomethylation of Cys was specified as the fixed modification, and oxidation on Met and acetylation on the protein N-terminus were specified as variable modifications. For the phosphoproteome analysis, Ser, Thr and Tyr phosphorylation were also set as variable modifications. False discovery rate (FDR) thresholds for protein, peptide and modification sites were specified at 1%. The minimum peptide length was set to 7 residues. All the other parameters in MaxQuant were set to the default values. The site localization probability was set to >0.5, and only sites whose localization probability was greater than 0.75 were considered confident and used in the subsequent analyses.

GO term association and enrichment analyses were performed with using the Database for Annotation, Visualization and Integrated Discovery (DAVID). The KEGG database was used to identify enriched pathways with the Functional Annotation Tool in DAVID compared to the background. The domain analysis was performed with the InterPro database using Functional Annotation Tool in DAVID. The manually curated CORUM protein complex database was used to analyse the protein complexed. The STRING database system was used. Functional protein-protein interaction networks were visualized using Cytoscape. When performing the bioinformatics analysis, a corrected p-value < 0.05 was considered significant.

### MTT assay

The cell growth curves were measured using 3-(4,5-dimethyl-2-thiazoyl)-2, 5-diphenyltetrazolium bromide (MTT) according to manufacturer’s instructions (Sigma). HT22 cells were seeded in 96-well plates at a density of 5,000 cells/well and treated with propofol or dexmedetomidine at the dose indicated in the figures for 8 hours. Then, 0.5 mg/mL MTT was added to each well, and the cells were incubated for 4 hours. After removing the media, 150 μL of DMSO were added to each well and the cells were agitated on an orbital shaker for 15 min. The optical density of the solubilized formazan crystals was then measured at 490 nm using a plate reader (Bio-Rad).

## Additional Information

**How to cite this article:** Zhang, H. *et al*. Quantitative analyses of the global proteome and phosphoproteome reveal the different impacts of propofol and dexmedetomidine on HT22 cells. *Sci. Rep.* **7**, 46455; doi: 10.1038/srep46455 (2017).

**Publisher's note:** Springer Nature remains neutral with regard to jurisdictional claims in published maps and institutional affiliations.

## Supplementary Material

Supplementary Information

Supplementary Table S1

Supplementary Table S2

Supplementary Table S3
