# Supplementary material for: Quantitative analyses of the global proteome and phosphoproteome reveal the different impacts of propofol and dexmedetomidine on HT22 cells
Source: Sci Rep. 2017 Apr 18;7:46455. doi: 10.1038/srep46455 (PMC5394455; doi:10.1038/srep46455)
Supplement: Supplementary Information [file srep46455-s1.pdf]

Supplementary Information for

Quantitative analysis of global proteome and phosphoproteome reveal the differential impacts of propofol and dexmedetomidine on HT22 cells

Honggang Zhang[1], Juan Ye[2], Zhaomei Shi[3], Chen Bu[3] and Fangping Bao[1*]

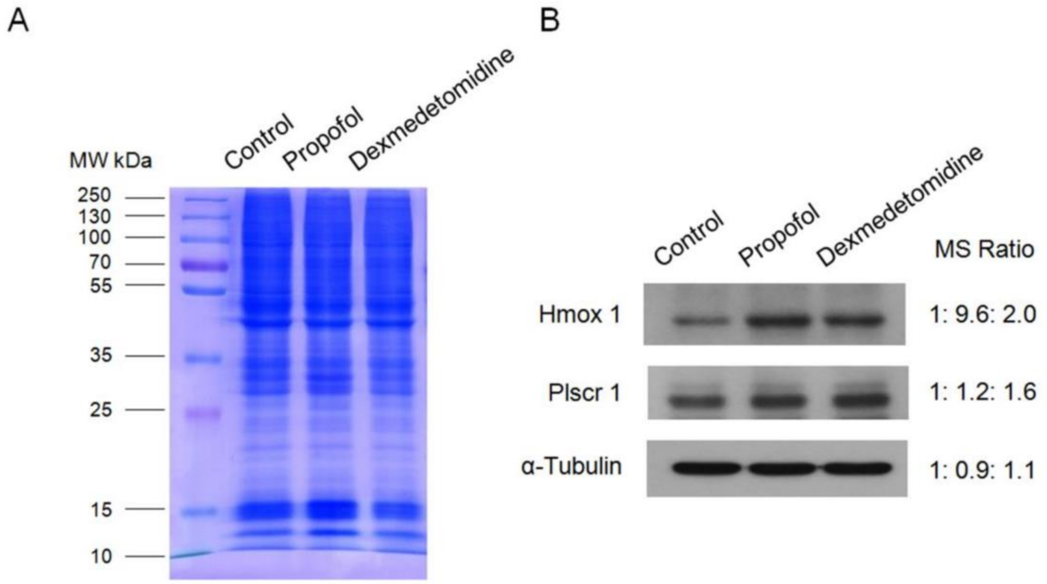

Figure S1 Validation of the proteome quantification. Proteins were extracted and then SDS-PAGE (A) and westernblots (B) were performed as indicate.

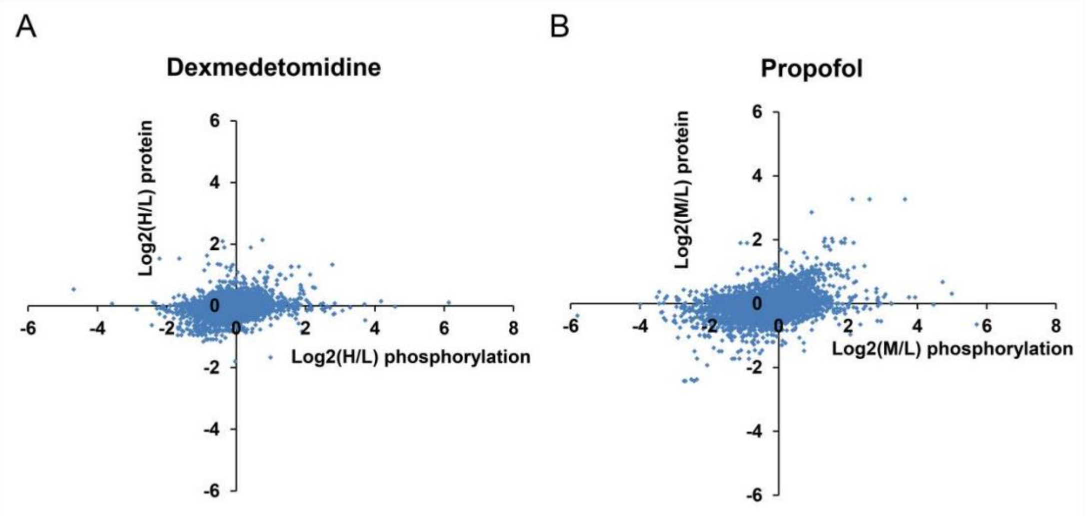

Figure S2 The correlation of quantitative ratios of proteome and phosphoproteome upon dexmedetomidine (A) and propofol (B) treatment.
